# Arctic cut-off high drives the poleward shift of a new Greenland melting record

**DOI:** 10.1038/ncomms11723

**Published:** 2016-06-09

**Authors:** M. Tedesco, T. Mote, X. Fettweis, E. Hanna, J. Jeyaratnam, J. F. Booth, R. Datta, K. Briggs

**Affiliations:** 1 Lamont-Doherty Earth Observatory, Columbia University, New York, New York 10964, USA; 2 NASA Goddard Institute of Space Studies, New York, New York 10025, USA; 3 University of Georgia, Athens, Georgia 30602-2502, USA; 4 University of Liege, Liege 4000, Belgium; 5 University of Sheffield, Sheffield S10 2TN, UK; 6 The City College of New York, New York, New York 10031, USA; 7 The Graduate Center of the City University of New York, New York, New York 10016, USA; 8 University of Leeds, Leeds LS2 9JT, UK

## Abstract

Large-scale atmospheric circulation controls the mass and energy balance of the Greenland ice sheet through its impact on radiative budget, runoff and accumulation. Here, using reanalysis data and the outputs of a regional climate model, we show that the persistence of an exceptional atmospheric ridge, centred over the Arctic Ocean, was responsible for a poleward shift of runoff, albedo and surface temperature records over the Greenland during the summer of 2015. New records of monthly mean zonal winds at 500 hPa and of the maximum latitude of ridge peaks of the 5,700±50 m isohypse over the Arctic were associated with the formation and persistency of a cutoff high. The unprecedented (1948–2015) and sustained atmospheric conditions promoted enhanced runoff, increased the surface temperatures and decreased the albedo in northern Greenland, while inhibiting melting in the south, where new melting records were set over the past decade.

## Abstract (web summary)

Atmospheric circulation controls the mass and energy balance of the Greenland ice sheet, yet the exact dynamics remain unknown. Here, the authors show that record conditions over Greenland during the summer of 2015 were associated with the formation and persistency of an Arctic cut-off high.

## Graphical abstract

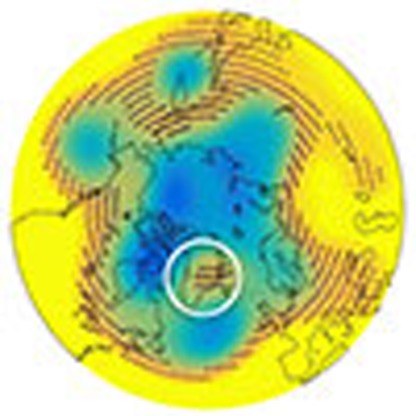

Atmospheric circulation affects the energy and mass budgets of the Greenland ice sheet^1^,^2^,^3^,^4^,^5^ by controlling cloud coverage and optical depth^6^, and by driving the spatial distribution, and amount of surface melting and accumulation^7^,^8^. Improving our understanding of the impact of atmospheric circulation on the Greenland's surface mass balance is, therefore, crucial for the refinement of climate and ice-sheet models, and will ultimately enable improved estimates of current and future contributions to sea level, by the largest ice body in the Northern Hemisphere.

Here we show that a poleward shift of melting record over the Greenland ice sheet in 2015 was driven by the exceptional atmospheric conditions characterized by new records in mean zonal winds and jet stream wave amplitude associated with the formation and evolution of a Arctic cutoff high.

## Results

### Atmospheric conditions and indicators

**Figure 1:**
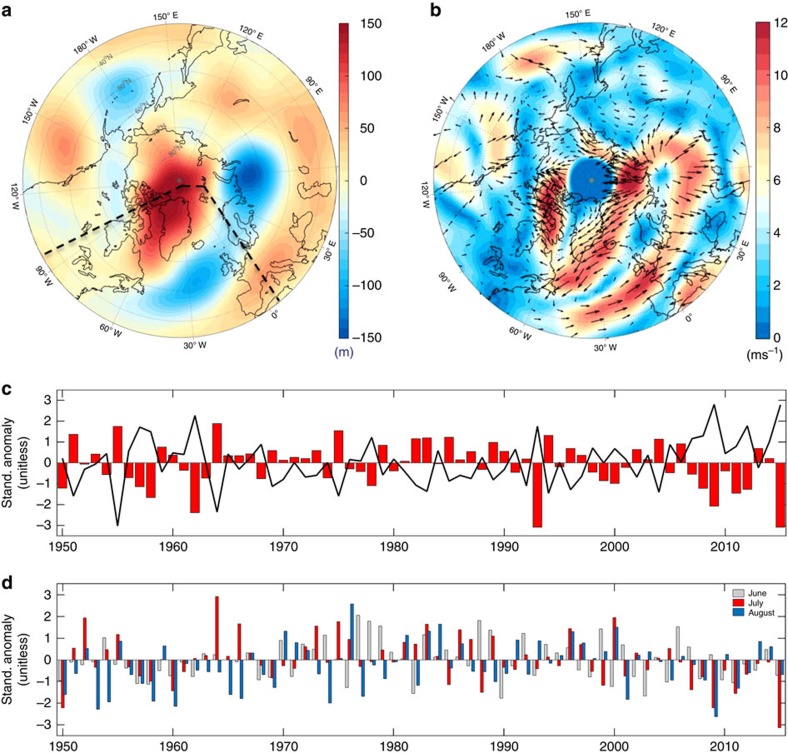
Atmospheric conditions and selected quantities over the Arctic and over the Greenland ice sheet. (**a**) 500 hPa geopotential height composite anomaly (m) for the month of July 2015, with respect to the 1981–2010 baseline period (using NCEP–NCARv1 reanalysis); (**b**) same as **a**, but for the vector winds (ms^−1^). (**c**) Time series of monthly averaged July NAO (red bars) and GBI (black line) indices (unitless) for the period 1950–2015. (**d**) Time series of standardized anomalies for the zonal winds at 500 hPa (unitless) averaged over the months of June (light gray), July (red) and August (pale blue) over the region bounded between 45–85° N and 100° W–0° E (included in the area marked by the dashed lines in **a**).

Our analysis of the geopotential height at 500 hPa (Methods) shows that during July 2015 a persistent atmospheric ridge was centred over the Arctic Ocean (Lincoln Sea, north of Greenland), with geopotential height anomalies being up to 3.7 s.d.'s (*σ*,∼150 m) above the 1981–2010 long-term mean (Fig. 1a). The North Atlantic Oscillation (NAO; Methods) and Greenland Blocking Index (GBI, defined as the 500 hPa geopotential height area averaged between 60–80° N and 20–80° W (ref. 9); Methods) have been associated with extreme melting events over the Greenland^9^,^10^,^11^,^12^. The summer average (June-July-August) value for NAO in 2015 of −1.61 was close to the summer value in 2012 of −1.59. Differently from 2015, however, the atmospheric ridge in 2012 was centred over the Greenland ice sheet^2^,^10^ (Supplementary Figs 1 and 2). The July monthly averaged NAO value set a new record low of −1.23 (since 1899), being 3.2*σ* below the 1981–2010 mean (Fig. 1c). Concurrently, the GBI also set a new record for the month of July (Fig. 1c; Supplementary Fig. 3b), being 2.8*σ* above the 1981–2010 mean. The June and August conditions in 2015 were not as exceptional in 2012, with mean June and August NAO values in 2015 being higher than the same quantities in 2012 (Supplementary Fig. 3a).

### Arctic cutoff high and new surface Greenland records

**Figure 2:**
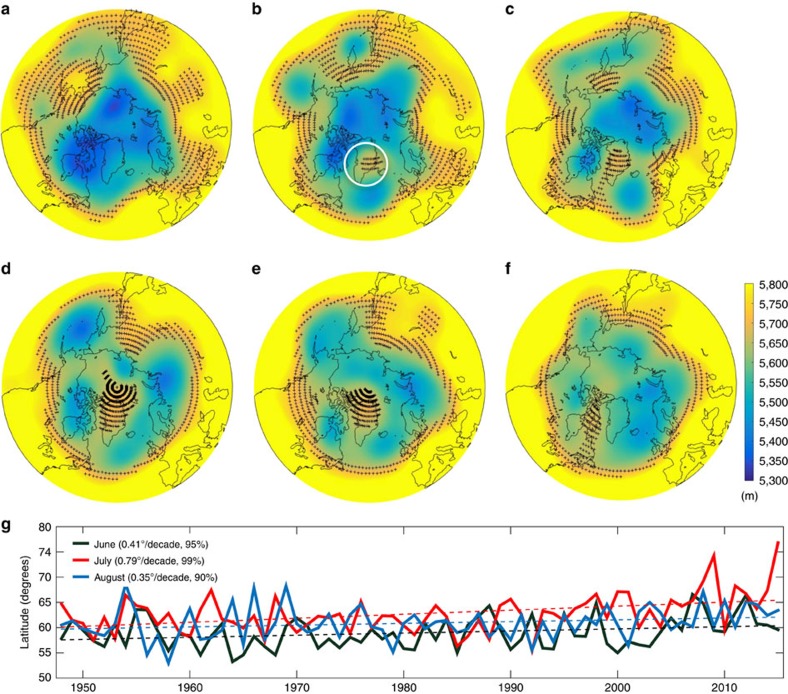
Spatial and temporal evolution of the jet stream conditions and wave amplitude. Jet stream is here characterized through the 5,700±50 m 500 hPa isoheights^13^. (**a**–**f**) Five day average geopotential height (m) at 500 hPa for the period 18th June to 22nd July 2015. For each day, the daily averaged values for the 2 days before and after were averaged with the daily average value of that day. Crosses show the locations where geopotential height values are 5,700±50 m. (**g**) Maximum latitude of ridge peaks computed from the 500 hPa 5,700±50 m isoheight for the period 1948–2015 over the region bounded between 45–85° N and 100° W–0° E (included in the area marked by the dashed lines in Fig. 1b), averaged over the months of June (black), July (red) and August (blue). Linear trends and significance levels are reported in the figure.

**Figure 3:**
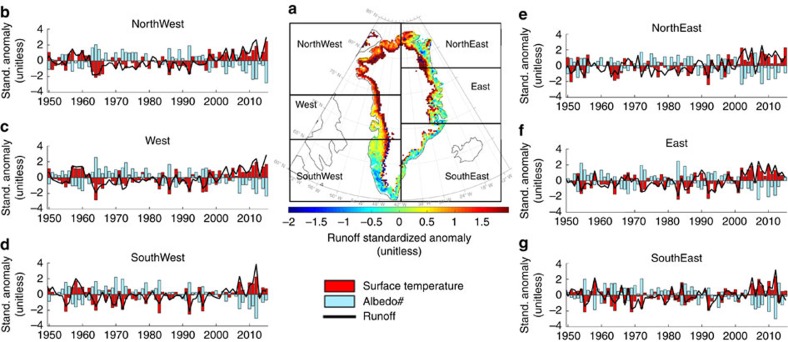
Surface mass balance and energy balance quantities over the Greenland ice sheet. (**a**) Spatial distribution of the July 2015 MAR-simulated runoff anomaly (unitless) over the Greenland ice sheet (1981–2010). The boxes in the map display the boundaries of the different drainage regions. (**b**–**g**) Time series of standardized anomalies (unitless) for mean July runoff (black line), surface temperature (red bars) and surface broadband albedo (cyan bars) for the period 1950–2015 over the different drainage basin regions identified in **a**, as simulated by the MAR model.

The July 2015 high-pressure ridge over the Greenland evolved from a cutoff high that formed along the eastern coast of Greenland at the end of June (white circle in Fig. 2b). Over the same period, the jet stream, here characterized through the 5,700±500 m 500 hPa isoheights^13^, broke into three positive heights around the Northern Hemisphere (Fig. 2c). These conditions reinforced the atmospheric ridge over the Greenland ice sheet, which moved westward and persisted until mid-July (Fig. 2b–e). This promoted new records for meltwater production, runoff, albedo and surface temperature over northwest Greenland (Fig. 3; Supplementary Fig. 4), as simulated by the Modèle Atmosphérique Régionale^1^,^3^,^7^ (MAR; Methods). The monthly averaged record setting values for simulated albedo and surface temperature in northwest Greenland for July 2015 were, respectively,∼2.5*σ* below and above the 1981–2010 mean, while runoff was up to ∼3*σ* above the mean (Fig. 3). The spatial distribution of the 2015 surface albedo anomaly simulated by MAR (Supplementary Fig. 5a–c) indicates that the July 2015 negative anomaly was driven by an albedo decrease at relatively high elevations, promoted by the reduced summer snowfall (associated with anticyclonic conditions) and by increased surface melting and runoff. The same atmospheric conditions that promoted these new records over the northern Greenland also inhibited melting in the south, where enhanced melting and new records have been occurring over the past recent years^1^,^10^. This had implications for the surface mass balance of Greenland at both regional and ice-sheet scales (Supplementary Fig. 6). The summer exposure of bare ice and the presence of surface impurities have been suggested to be driving the enhanced melting observed over the past ∼20 years^1^. However, the 2015 summer atmospheric conditions promoted the flow of cold air from the Arctic Ocean (Fig. 1b), favouring the accumulation of fresh new snow with a high albedo along western Greenland, hence offsetting the effects of bare ice exposure (Supplementary Figs 5–7).

### Mean zonal winds and jet stream wave amplitude records

The westward shift of the cutoff high between the end of June and the beginning of July 2015 is associated with new records for both the mean zonal winds at 500 hPa (Fig. 1d) and the maximum latitude of ridging (Fig. 2g) over the region bounded between 45–85° N and 100° W–0° E. The July-averaged value of the 500 hPa zonal winds speed over the Greenland for latitudes between 60° and 80° N was −1.5 m s^−1^ (here, the negative sign indicates easterly flow), ∼3*σ* below the mean. The two other occurrences of easterly flow for the same quantity during the 1948–2015 period happened in 1950 and 2009. However, the mean wind speed values were only ∼−0.15 m s^−1^ in both cases, hence much smaller in magnitude than the 2015 record value. The monthly mean maximum latitude of ridge peaks of the 5,700±50 m isohypse over the Atlantic sector (45–85° N and 100° W–0° E) also set the new record of 76.61° N (3.4*σ*) in July 2015, exceeding the previous record of 74.92° N (2.9*σ*) set in 2009 (Fig. 2g). The trend for the maximum latitude of the ridge peaks for the period 1948–2015 for the month of July is 0.79±0.09° per decade. The same trends for the months of June and August are, respectively, 0.41±0.14° per decade and 0.35±0.19° per decade. Such trends are even larger when considering only the satellite era (1979–2015), being up to ∼2.4° per decade for the month of July and are confirmed by the analysis of different global atmospheric reanalysis data sets (Supplementary Fig. 8).

## Discussion

The mechanisms that created and maintained the 2015 observed ridge may be linked with forcing from very strong extratropical cyclones^14^, to forcings from southern regions^15^ or to latent heat release^16^. Another possibility is the local forcing related to Arctic amplification^13^,^17^. Although recent melt records over the Greenland have been linked to exceptional mid-tropospheric atmospheric conditions, with episodes of atmospheric blocking ridges being associated with Greenland's melting extremes^9^,^12^, little or no attention has been given to the impact of the anticipated effects of Arctic amplification on the surface mass balance of the Greenland ice sheet. In this regard, the 2015 records for both the 500 hPa zonal winds and the maximum ridging latitude are consistent with the proposed effects on upper level atmosphere characteristics associated with Arctic amplification^13^,^17^.

The 2015 poleward shift of the surface melting record in 2015, clearly indicates that improving our understanding of the impact of exceptional atmospheric conditions on the spatial distribution of extreme melting is crucial. Besides modulating the contribution of Greenland to sea level through the volume of meltwater production, the location of enhanced melting can influence ocean/ice interaction processes and ocean circulation^18^, and bio-productivity, by altering salinity and temperature profiles of the surrounding ocean. Furthermore, the evolution of surface melting strongly impacts the Greenland's hydrological system, with implications for the englacial and subglacial systems, as well as ice discharge and dynamics^19^. Currently, several general circulation global climate models and Earth System models do not properly capture summer Arctic atmospheric forcing^8^, limiting our capability to properly project the evolution of the surface mass balance and melting under future warming scenarios. Our work presented here demonstrates a strong need to identify the mechanisms that create and maintain strong cutoff highs. The new atmospheric records, and the trends of mean zonal winds and wave amplitude of the jet stream are consistent with the suggested effects of Arctic amplification^13^,^17^. Recent studies provide theoretical arguments that slowing zonal winds might be associated with larger planetary wave amplitudes^20^ and that Arctic amplification and/or sea-ice loss do intensify existing ridges, thereby contributing to their persistence^21^,^22^. In the event studied here, however, the exceptional melting followed the ridging, rather than preceding it in alignment with other studies, indicating that observations and models results do not support the above mentioned expected effects of Arctic amplification^23^,^24^,^25^,^26^,^27^. Be that as it may, understanding the impact of cutoff highs on the Greenland's surface mass balance, and studying the mechanisms driving the trends and extremes of the anticipated effects of Arctic amplification are crucial tasks in view of the potential regional and global impacts long-time effects of enhanced melting over Greenland.

## Methods

### NAO data set

For the NAO index, we used the Hurrell^28^ NAO values distributed by the Climate Research Unit of the University of Anglia ( http://www.cru.uea.ac.uk/cru/data/nao/). The Hurrell NAO index is computed from the difference of normalized sea-level pressure between Lisbon, Portugal and Stykkisholmur/Reykjavik, and Iceland. More detail on the Hurrell NAO values can be found at https://climatedataguide.ucar.edu/climate-data/hurrell-north-atlantic-oscillation-nao-index-pc-based (ref. 28).

### Reanalysis data

Geopotential heights and zonal winds at 500 hPa are obtained from the National Centers for Environmental Prediction (NCEP)/National Center for Atmospheric Research (NCEP/NCAR) reanalysis data set. The NCEP/NCAR data set consists of globally, gridded data sets, incorporating observations and outputs from a numerical weather prediction model from 1948 to present^29^. Values of the GBI are computed from the daily and monthly values of the NCEP/NCAR 500 hPa geopotential height values averaged over the area between 60–80° N and 280–340° E (ref. 9). ERA-Interim reanalysis data is used to complement the results obtained with NCEP/NCAR and was downloaded from http://apps.ecmwf.int/datasets/data/interim-full-daily/levtype=sfc/.

### The MAR regional climate model

Simulations of surface quantities over the Greenland ice sheet are performed using the MAR^1^,^7^,^8^ MAR is a modular, hydrostatic, and compressible atmospheric model that uses the sigma-vertical coordinate to better simulate airflow over complex terrain and the Soil Ice Snow Vegetation Atmosphere Transfer scheme surface model. The snow model in MAR is the CROCUS model^30^. The MAR model configuration used here has 25 terrain-following sigma layers between the Earth's surface and the top. The horizontal resolution of the outputs used here is 20 km. The lateral boundary conditions are prescribed every 6 h from NCEP/NCAR meteorological fields. Here we use daily outputs obtained from the average of 120 s outputs produced by the model. The temporal configuration for the runs is from 1948 to the present. The sea surface temperature and the sea-ice cover are also prescribed every 6 h in the model, using NCEP/NCAR reanalysis data^29^. No nudging or interactive nesting is used in any of the experiments, with the atmospheric fields over the Greenland ice sheet computed by the atmospheric module of MAR. The atmospheric model, in turn, interacts with the CROCUS model, which provides the state of the snowpack, and associated surface mass balance and energy balance quantities (for example, albedo and runoff).

### Code availability

MAR is an open-source code available to the scientific community. The source code for the MAR version used in this study is available at ftp://tedesco-dell.ldeo.columbia.edu/cryoftp/MARv3.5.2src_2015-03-18.tgz. The codes used for analysing the reanalysis data are available upon request from the authors.

### Data availability

MAR outputs referenced in this study are publicly available at ftp://tedesco-dell.ldeo.columbia.edu/cryoftp/. ERA-Interim data is available at http://apps.ecmwf.int/datasets/data/interim-full-daily/levtype=sfc/. NCEP data is available at http://www.esrl.noaa.gov/psd/data/gridded/data.ncep.reanalysis.html.

## Additional information

**How to cite this article:** Tedesco, M. *et al*. Arctic cut-off high drives the poleward shift of a new Greenland melting record. *Nat. Commun.* 7:11723 doi: 10.1038/ncomms11723 (2016).

## Supplementary Material

Supplementary Information Supplementary Figures 1-8
