# Supplementary material for: Arctic cut-off high drives the poleward shift of a new Greenland melting record
Source: Nat Commun. 2016 Jun 9;7:11723. doi: 10.1038/ncomms11723 (PMC4906163; doi:10.1038/ncomms11723)
Supplement: Supplementary Information — Supplementary Figures 1-8 [file ncomms11723-s1.pdf]

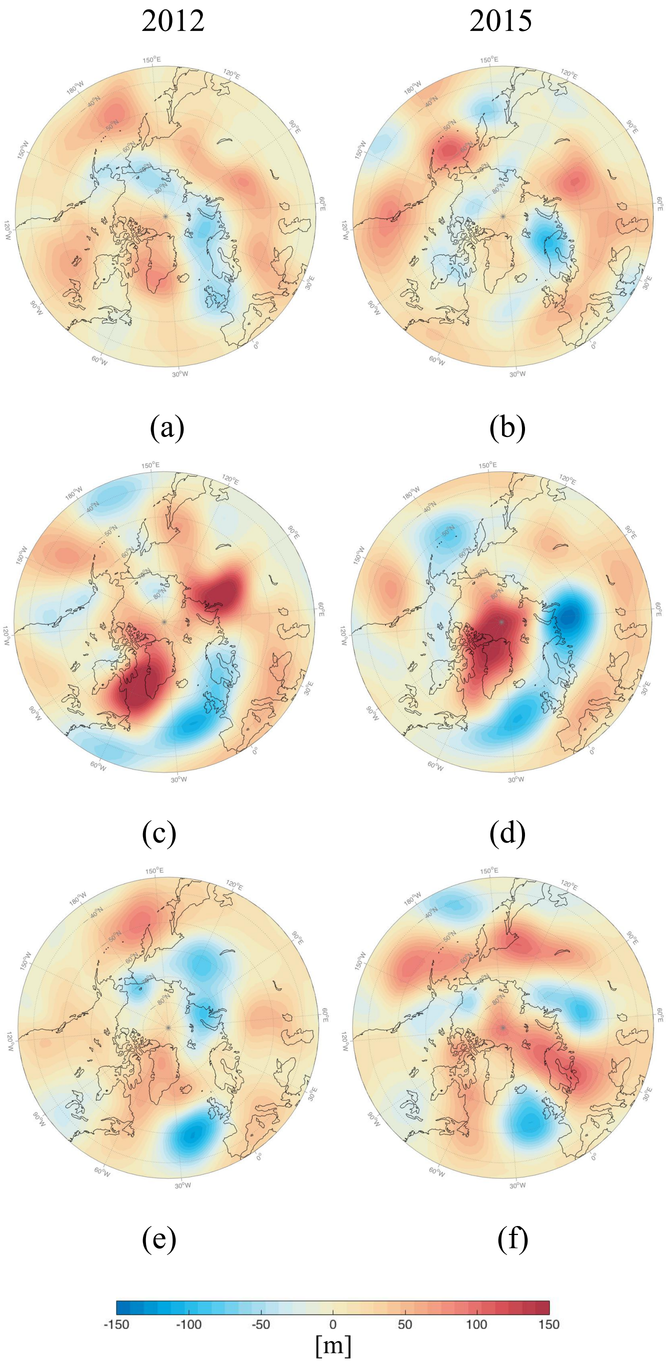

Supplementary figure 1. Monthly-averaged geopotential height 500hPa anomalies (1981 – 2010) expressed in meters for the months of June (a,b), July (c,d) and august (e,f) for the years 2012 (left column) and 2015 (right column).

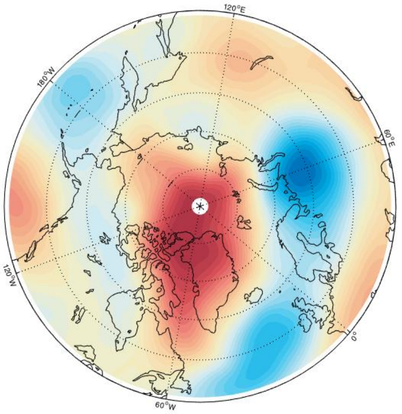

(a)

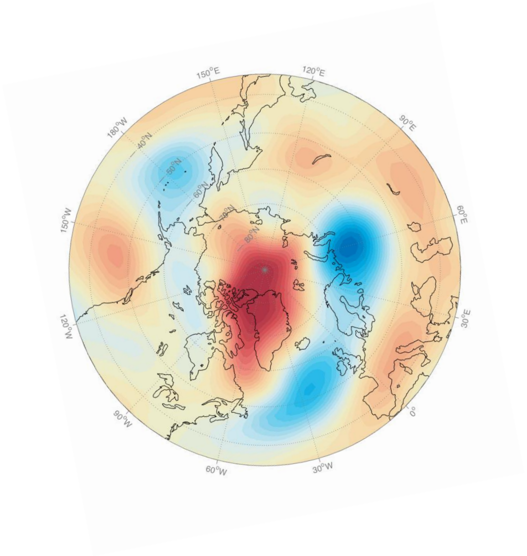

(b)

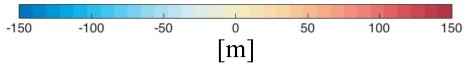

Supplementary figure 2. Monthly-averaged geopotential height 500hPa anomalies (1981 – 2010) expressed in meters for the month of July for 2015 obtained from the a) ERA-Interim and b) NCEP/NCAR reanalysis datasets.

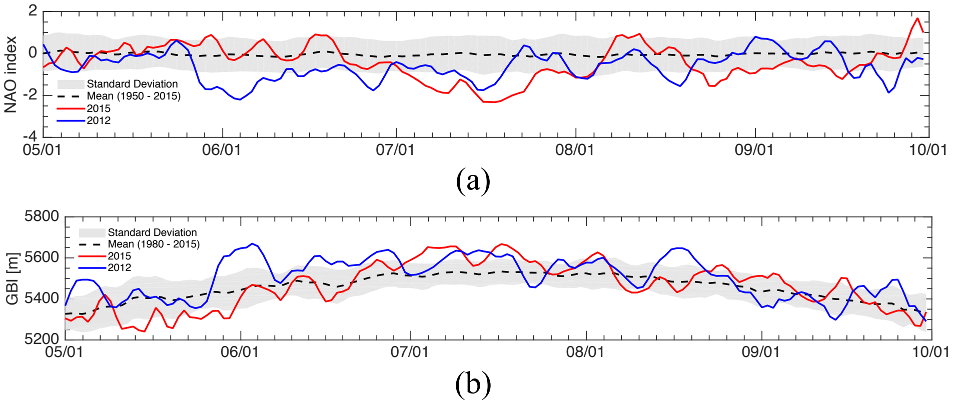

Supplementary figure 3. Daily a) NAO and b) GBI index values for the period May 1 – October 1 for years 2015 (red) and 2012 (blue). The dashed black line represents the 1981 – 2010 averaged values and the gray-shaded area represents ±1 standard deviation from the mean.

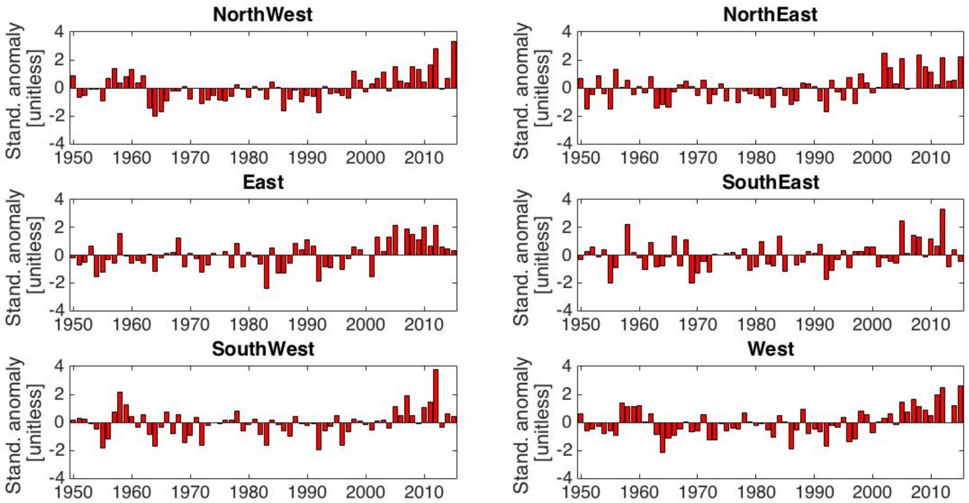

Supplementary figure 4. Time series of standardized anomalies [unitless] for mean July meltwater production for the period 1950 – 2015 over the different drainage basin regions identified in Figure 3 as simulated by the MAR model forced by NCEP/NCAR re-analysis data.

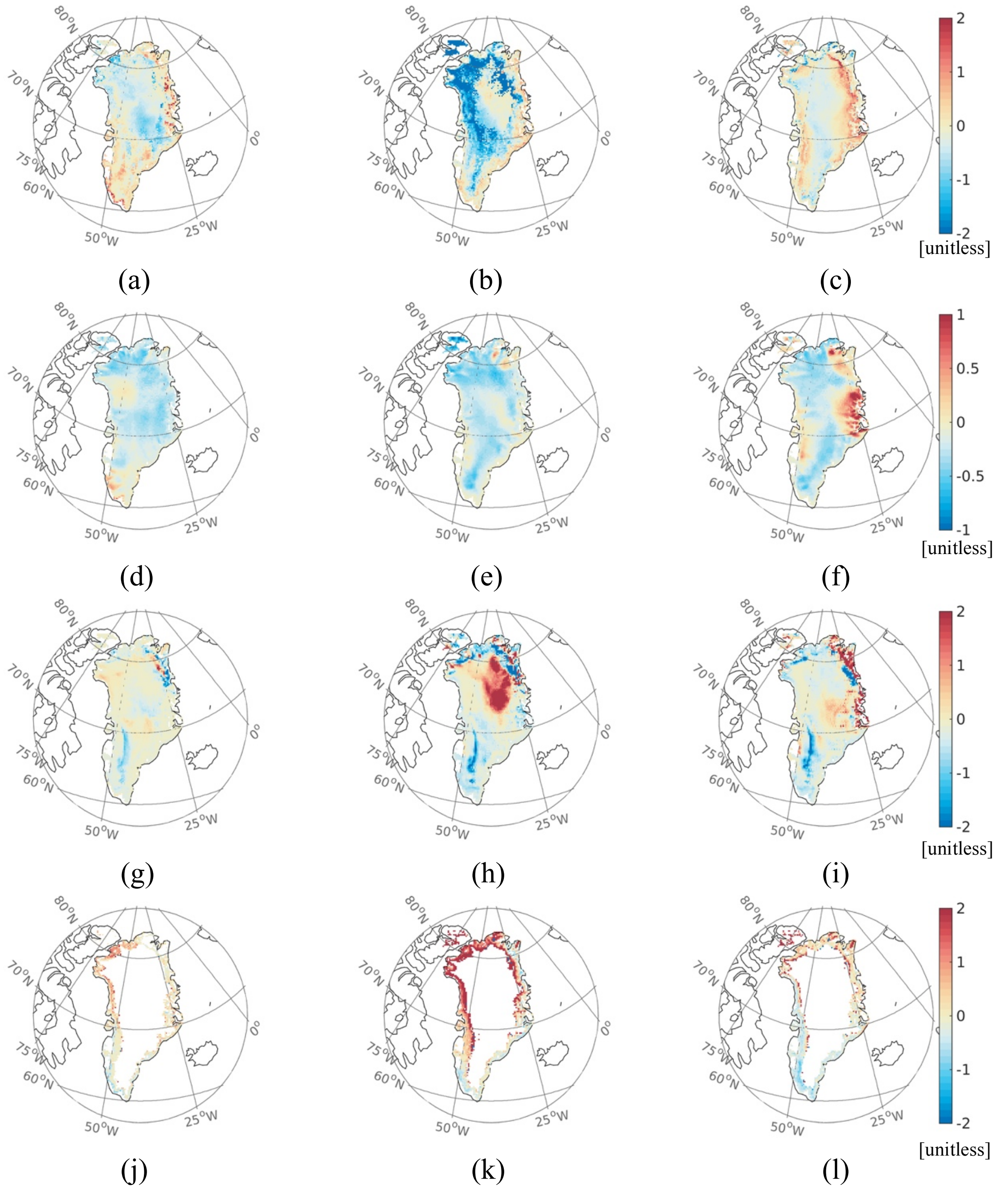

Supplementary figure 5. Anomaly maps of monthly averaged values for standardized anomalies of albedo (a – c), snowfall (d – f), rainfall (g – i) and runoff (j – l) for the months of June (left column), July (mid column) and August (right column), as simulated by MAR forced by NCEP-NCARv1 reanalysis. Baseline period to compute anomalies is 1981 – 2010.

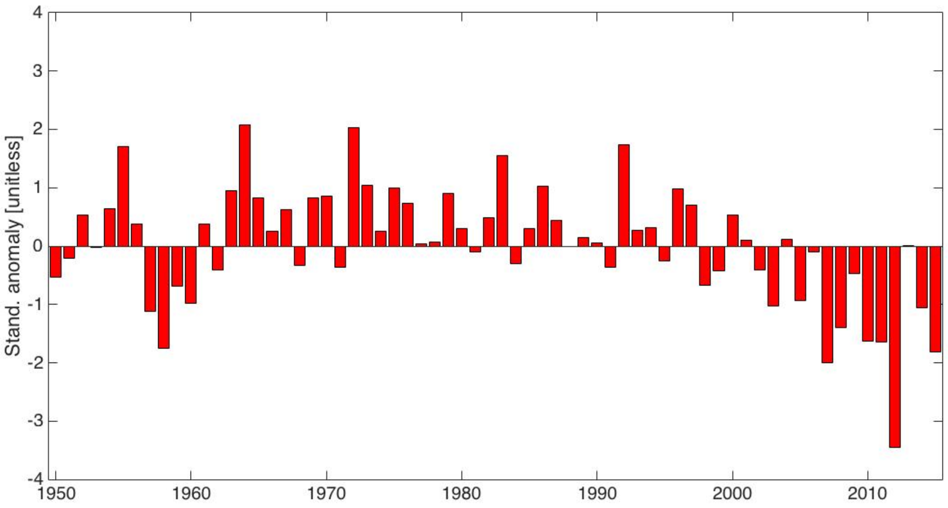

(a)

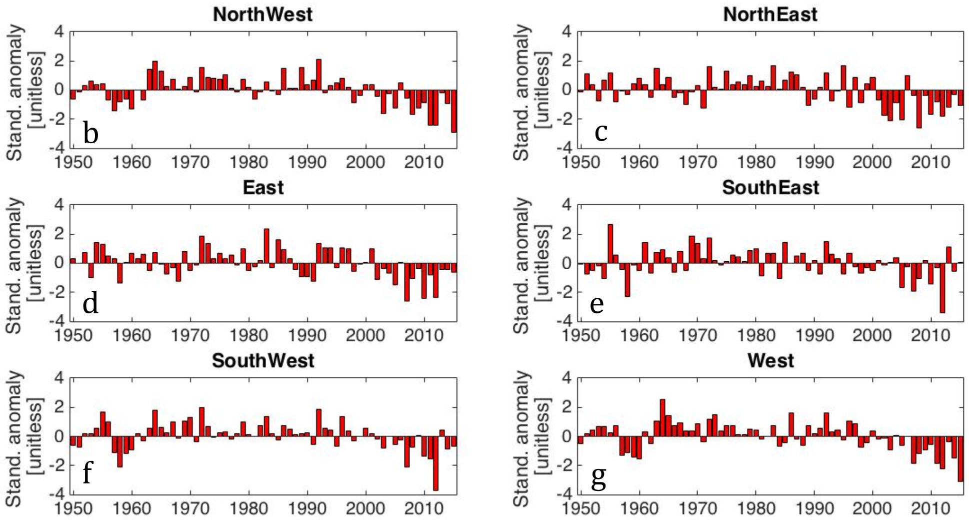

Supplementary figure 6. Time series of standardized anomalies [unitless] for mean July surface mass balance for the period 1950 – 2015 over a) the whole ice sheet and b-g) over the different drainage basin regions identified in Figure 3 as simulated by the MAR model forced by NCEP/NCAR re-analysis data.

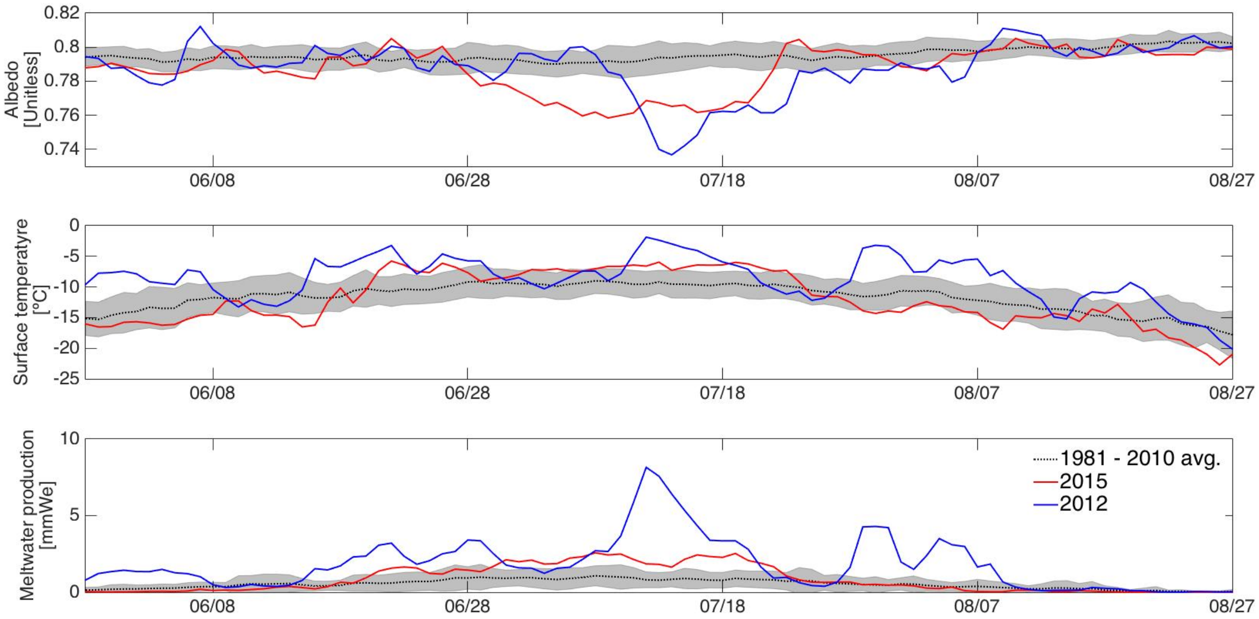

Supplementary figure 7. Daily a) albedo [untiless], b) surface temperature [ºC] and c) meltwater production [mmWe] for the period June 1 – September 1 for years 2015 (red) and 2012 (blue) as simulated by MAR forced with NCEP-NCARv1 reanalysis. The dashed black line represents the 1981 – 2010 averaged values and the gray shaded area represents ±1 standard deviation from the mean.

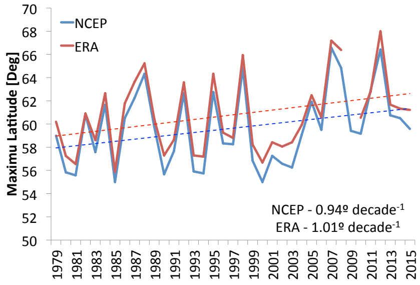

(a)

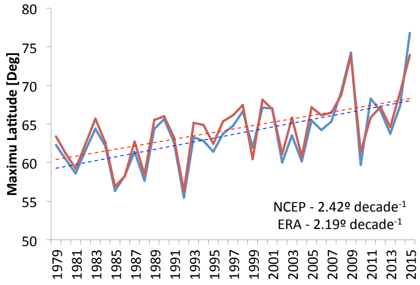

(b)

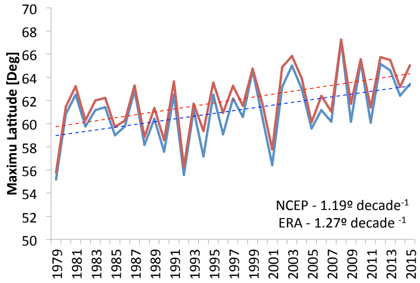

(c)

Supplementary figure 8. Maximum latitude of ridge peaks computed from the 500 hPa 5700±50 m isoheight for the period 1979 – 2015 over the region bounded between 45–85ºN and 100ºW – 0ºE (included in the area marked by the dashed lines in Fig. 1a), averaged over the months of a) June, b) July and c) August using the NCEP-NCAR (Kalnay et al. 1996, blue coloured lines) and ERA-Interim (http://www.ecmwf.int/en/research/climate-reanalysis/era-interim) datasets. Linear trends for the results obtained with the two reanalysis products are report in the lower left corner of each panel.
